# Systematic Analysis of the Clinical Significance of Hyaluronan-Mediated Motility Receptor in Colorectal Cancer

**DOI:** 10.3389/fmolb.2021.733271

**Published:** 2021-10-26

**Authors:** Yan-ping Tang, Yi-xin Yin, Ming-zhi Xie, Xin-qiang Liang, Ji-lin Li, Ke-zhi Li, Bang-li Hu

**Affiliations:** ^1^ Department of Research, Guangxi Medical University Cancer Hospital, Nanning, China; ^2^ Department of Chemotherapy, Guangxi Medical University Cancer Hospital, Nanning, China

**Keywords:** colorectal cancer, hyaluronan-mediated motility receptor, tissues, blood, cell lines

## Abstract

**Background:** The role of hyaluronan-mediated motility receptor (*HMMR*) in colorectal cancer (CRC) remains unclear. The present study aimed to explore the association of *HMMR* with the development and prognosis of CRC using sequence datasets, clinical tissues, blood samples, and cell lines.

**Methods:** CRC datasets were downloaded from TCGA and GEO databases. Forty CRC tissue samples, 120 CRC blood samples, and 100 healthy controls were collected. Four CRC cell lines (HCT116, HT-29, LoVo, and SW480) and one normal human colon mucosal epithelial cell line (NCM460) were cultured. RT-qPCR was used to determine the expression of *HMMR* in the tissues and cell lines. ELISA was used to measure *HMMR* levels in the blood samples.

**Results:** The expression of *HMMR* was significantly increased in CRC tissues than in corresponding adjacent tissues based on TCGA and GEO datasets, and clinical CRC tissues. No associations were found between the expression of *HMMR* and the TNM stage or other clinical parameters. The expression of *HMMR* varied in different CRC cell lines. The blood levels of *HMMR* tended to be higher in patients with CRC than in healthy controls. TCGA and GEO datasets showed inconsistent results regarding the association of *HMMR* expression with the survival of patients with CRC.

**Conclusion:** The expression of *HMMR* is increased in CRC tissues but not in the blood. The expression of *HMMR* is independent of CRC development and has no prognostic significance in patients with CRC.

## Introduction

Colorectal cancer (CRC) is one of the most common malignancies worldwide, according to the GLOBOCAN 2020 reports (Sung et al., 2021). Despite advances in surgical techniques, and adjuvant and neoadjuvant therapies, the prognosis of patients with CRC at a later stage remains poor. The pathogenesis of CRC is a multifactorial process, and molecular changes are one of the crucial factors that lead to the development of CRC. Several molecules are associated with the development and progression of CRC, and some of them can serve as indicators of CRC diagnosis, treatment monitoring, and prognosis (Di Nicolantonio et al., 2021). However, other molecules still need to be explored.

The hyaluronan-mediated motility receptor (*HMMR*, also known as *RHAMM*), a hyaluronan receptor, is localized in the cytoplasm, nucleus, and cell surface and regulates cell motility and cell cycle (Evanko and Wight, 1999; Jiang et al., 2013; Carvalho et al., 2021). Evidence suggests that the *HMMR* is involved in the occurrence and development of tumors that are dependent on hyaluronan-mediated signaling (Tolg et al., 2003; He et al., 2020). Previous studies indicate that the *HMMR* is overexpressed in and is a potential prognostic factor in various cancer types, including breast cancer (Liu et al., 2016), head and neck squamous cell carcinoma (Lu et al., 2021), hepatocellular carcinoma (Lu et al., 2020), and other cancers.

Several studies have reported the role of *HMMR* in CRC. Zlobec et al. (Zlobec et al., 2008c) reported that high expression of *HMMR* was significantly associated with the five-year survival rate in patients with CRC. Sun et al. (Sun et al., 2020) analyzed data from The Cancer Genome Atlas (TCGA) and Gene Expression Omnibus (GEO) databases and found that the *HMMR* was significantly associated with overall survival in patients with CRC. The *HMMR* was also found to involve in the metastasis and treatment response in CRC (Lugli et al., 2006; Mele et al., 2017; Burren et al., 2021). These results indicate that the HMMR is a predictor of CRC in patients. However, due to limited data, the robustness of the results is undermined. Thus, the role of *HMMR* in CRC needs to be further elucidated. In the present study, we explored the role of *HMMR* in CRC by comprehensively analyzing its expression in CRC sequencing databases, tissue and serum samples, and cell lines; this study may provide more reliable results to validate the role of HMMR in CRC.

## Materials and Methods

### *HMMR* Data in CRC From TCGA and GEO Databases

TCGA data of HMMR in colon adenocarcinoma (COAD) and rectal adenocarcinoma (READ) were downloaded from the UCSC Xena database (http://xena.ucsc.edu/) that includes 440 colon cancer and 158 rectal cancer samples. The *HMMR* values and phenotypes of patients were extracted together. Twelve CRC microarray datasets were downloaded from the GEO database with accession numbers GSE21510 (num. 148), GSE21815 (num. 141), GSE31279 (num. 110), GSE44076 (num. 246), GSE32323 (num. 44), GSE113513 (num. 28), GSE164191 (num. 121), GSE47756 (num. 93), GSE17537 (num. 55), GSE12945 (num. 62), GSE17536 (num. 177), and GSE14333 (num. 290). Of these, GSE164191 and GSE47756 provided data from the blood of patients with CRC, and the remaining samples were from CRC tissues.

### Clinical CRC Sample Collection

We collected 40 CRC tissues and corresponding adjacent normal tissues from the BioBank of the Guangxi Medical University Cancer Hospital between January 2017 and December 2019. Fresh tissue samples were frozen immediately after surgery and stored in liquid nitrogen until further use. All tissues were histologically confirmed to be CRC. In addition, the blood samples of 120 CRC patients and 100 healthy controls were collected. The patients with CRC did not receive chemotherapy or other treatments prior to surgery, or suffer from diseases, such as immune diseases and severe major organ dysfunction. This study was approved by the Ethics Committee of the Guangxi Medical University Cancer Hospital (2021-KY-043), and written informed consent was obtained from all participants.

### Cell Lines and Culture

Four CRC cell lines, HCT116, LoVo, SW480, and HT-29, and one human colon mucosal epithelial cell line, NCM460, were obtained from the Chinese Academy of Sciences (Shanghai, China). HCT116, LoVo, SW480, and HT-29 cells were cultured in DMEM with GlutaMAX (Gibco) supplemented with 10% FBS and 1% streptomycin/penicillin. NCM460 cells were cultured in phenol red–free RPMI 1640 (Gibco) supplemented with 10% FBS and 1% streptomycin/penicillin. The identity of the cell lines was confirmed by STR profiling.

### RNA Isolation From Colon Cancer Tissues and Cells

Total RNA from CRC tissues and cell lines was isolated using TRIzol reagent (Invitrogen; Thermo Fisher Scientific, Inc., Waltham, MA, United States), according to the manufacturer’s instructions. The RNA concentration was tested using NanoDrop ND-1000, and the quality was assessed using electrophoresis with 1.5% denaturing agarose gels.

### RT-qPCR Procedure

First-strand cDNA was synthesized by reverse transcription from 1 µg RNA using the PrimeScript RT reagent Kit with gDNA Eraser (Takara, Dalian, China). qPCR was performed using the standard protocol from the SYBR^®^ Premix Ex Taq kit (Takara, Dalian, China), according to the manufacturer’s instructions. *GAPDH* was used as an internal control. The following primers were used: *HMMR*: forward, 5′-GCA GAA CCA ACT CAA GCA ACA G-3′, and reverse, 5′-TCT TCA TAG AGG AGA CGC CAC T-3'; *GAPDH*: forward, 5′-GCA CCG TCA AGG CTG AGA AC-3′, and reverse, 5′-TGG TGA AGA CGC CAG TGG A-3'. The expression of *HMMR* was quantified using the 2^−ΔΔCT^ method.

### Enzyme-Linked Immunosorbent Assay (ELISA) for *HMMR*

A 5 ml fasting peripheral blood sample was collected from each subject. Serum samples were obtained by centrifuging at 1,500 ×*g* for 10 min at 4°C and then stored at −80°C for further use. Serum samples from healthy controls were collected in the morning. The ELISA kit (Signalway Antibody, California, United States, No. EK4982) was used to measure serum HMMR levels. The experiments were performed according to the manufacturer’s instructions.

### Statistical Analysis

Data are presented as mean ± standard deviation. The χ^2^ test was used to compare differences in categorical variables, and Student’s *t*-test was used to compare differences between two groups for continuous variables. The one-way ANOVA test was employed to compare the continuous variables for over three groups. Kaplan–Meier survival curves were applied to depict the survival of patients, and the log-rank test was used to compare the survival rates. Statistical analyses were performed using R (version 3.4.1). A *p*-value < 0.05 was considered to be statistically significant.

## Results

### Expression of *HMMR* in Cancers in TCGA and GEO Datasets

**FIGURE 1:**
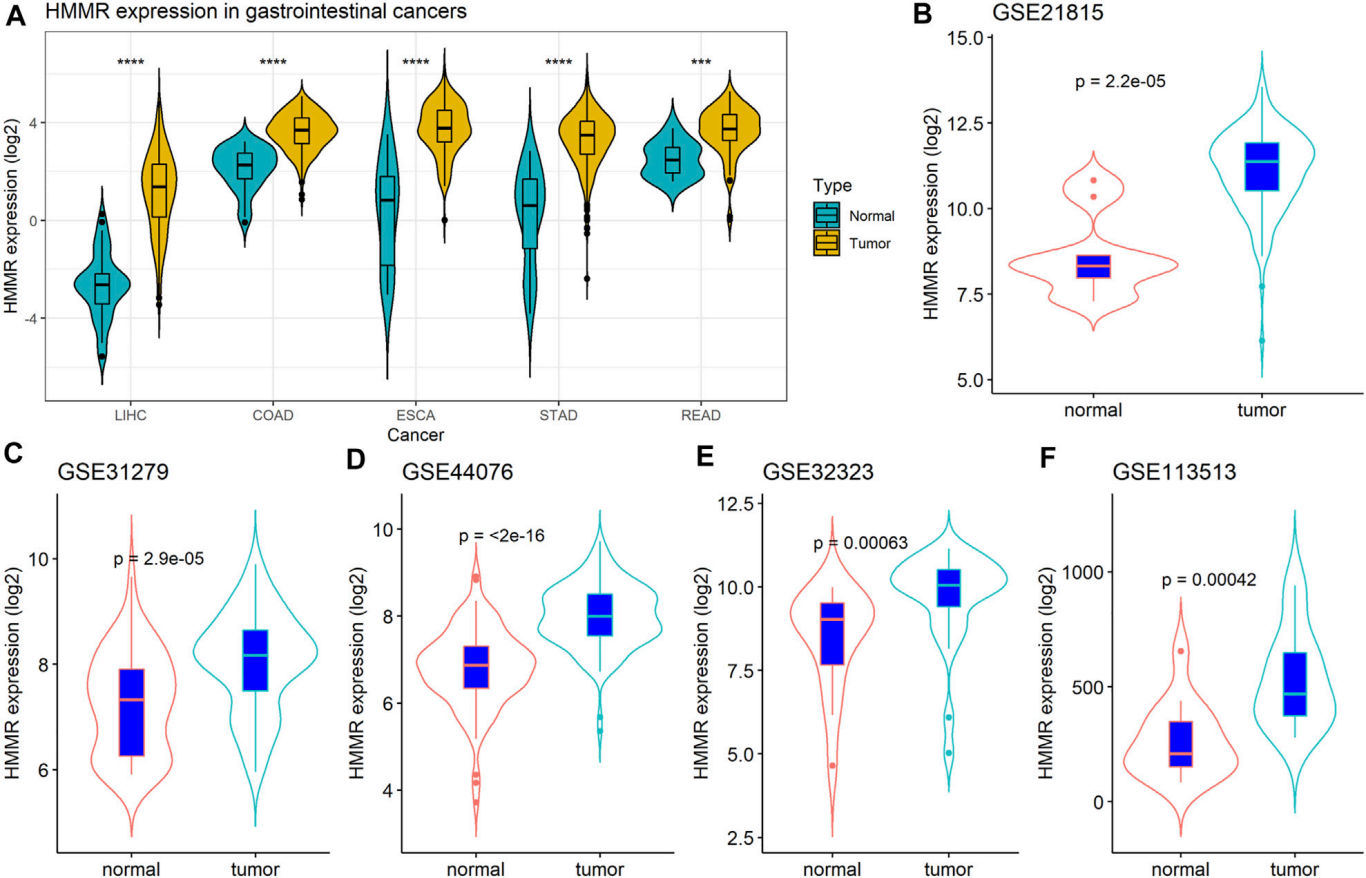
**(A)** Expression of *HMMR* in colorectal cancer (CRC) based on The Cancer Genome Atlas (TCGA) and Gene Expression Omnibus (GEO) datasets. **(B-F)** Comparison of HMMR in CRC and normal tissues in five GEO datasets.

We first analyzed the expression of *HMMR* mRNA in gastrointestinal cancers in TCGA, including colon cancer (COAD), rectal cancer (READ), liver cancer (LIHC), gastric cancer (STAD), and esophageal cancer (ESCA) datasets, and found that *HMMR* mRNA was significantly overexpressed in gastrointestinal cancer tissues than in normal tissues (Figure 1A, *p* < 0.05). Furthermore, we analyzed the expression of *HMMR* mRNA in GSE21815, GSE31279, GSE44076, GSE32323, and GSE113513 datasets and found that the expression of *HMMR* was significantly increased in the CRC tissues than in normal tissues (Figures 1B–F, *p* < 0.05).

### Validation of *HMMR* in Clinical Colon Cancer Tissues

**FIGURE 2:**
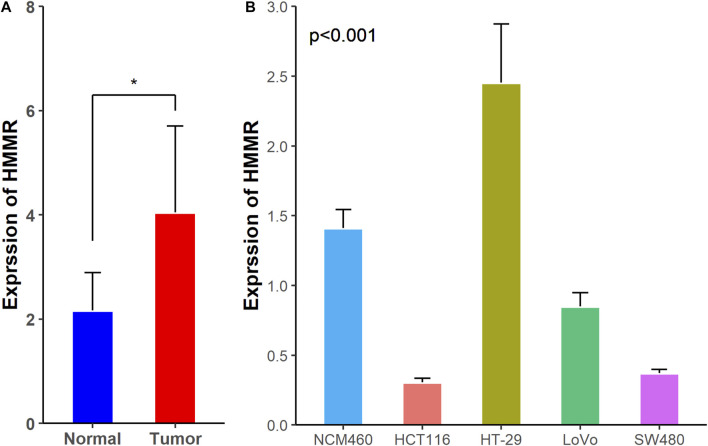
Expression of *HMMR* mRNA in **(A)** clinical tissues and **(B)** CRC cell lines and colon mucosal epithelial cell line. *p < 0.05.

Next, we checked the expression of *HMMR* in 40 colon cancer tissues and corresponding adjacent colon tissues by RT-qPCR. The analysis suggested that *HMMR* mRNA was significantly overexpressed in colon cancer tissues than in adjacent colon tissues (Figure 2A, *p* < 0.05), which verified the results from the online datasets.

### Expression of *HMMR* in Colon Cancer Cells

We checked the expression of *HMMR* mRNA in HCT116, HT-29, LoVo, SW480, and NCM460 using the RT-qPCR assay. The expression of *HMMR* mRNA was significantly increased in HT-29 cells, but decreased in HCT116, LoVo, and SW480 cells, especially in the HCT116 cells, than in NCM460 cells (Figure 2B).

### Clinical Significance of *HMMR* in CRC

We analyzed the clinical significance of HMMR in CRC using TCGA dataset, GEO dataset (GSE21815), and clinical datasets. The median HMMR level in CRC tissues was calculated and used as the cutoff value. As listed in Table 1, the expression of HMMR in CRC tissues was not associated with the patients’ age, sex, TNM stage, clinical stage, tumor location, and *KRAS* mutation status (*p* > 0.05), indicating that the expression of *HMMR* in CRC tissues was independent of these clinical parameters.

**TABLE 1:** Results of clinical significance of HMMR in CRC using three datasets.

	TCGA-COAD and -READ datasets			GSE21815 dataset			Clinical tissues		
—	High (299)	Low (299)	*p*-Value	High (66)	Low (66)	*p*-Value	High (20)	Low (20)	*p*-Value
Age	66.3 ± 12.6	66.3 ± 12.6	0.999	64.1 ± 12.8	65.1 ± 10.2	0.614	59.5 ± 12.3	57.9 ± 12.3	0.664
Gender	—	—	0.354	—	—	0.599	—	—	0.479
** **Female	129 (44.0)	144 (48.2)	—	31 (47.0)	27 (40.9)	—	4 (20.0)	7 (35.0)	—
** **Male	164 (56.0)	155 (51.8)	—	35 (53.0)	39 (59.1)	—	16 (80.0)	13 (65.0)	—
Location	—	—	0.926	—	—	—	—	—	0.715
** **Colon	219 (73.2)	221 (73.9)	—	—	—	—	16 (80.0)	14 (70.0)	—
** **Rectal	80 (26.8)	78 (26.1)	—	—	—	—	4 (20.0)	6 (30.0)	—
T stage	—	—	0.804	—	—	0.294	—	—	0.092
** **T1	10 (3.41)	10 (3.34)	—	1 (1.52)	5 (7.58)	—	0	0	—
** **T2	48 (16.4)	54 (18.1)	—	11 (16.7)	9 (13.6)	—	5 (25.0)	1 (5.00)	—
** **T3	207 (70.6)	200 (66.9)	—	38 (57.6)	41 (62.1)	—	9 (45.0)	7 (35.0)	—
** **T4	28 (9.56)	34 (11.4)	—	16 (24.2)	11 (16.7)	—	6 (30.0)	12 (60.0)	—
N stage	—	—	0.030	—	—	0.793	—	—	0.609
** **N0	173 (59.0)	163 (54.5)	—	36 (54.5)	35 (53.0)	—	8 (40.0)	7 (35.0)	—
** **N1	76 (25.9)	68 (22.7)	—	18 (27.3)	16 (24.2)	—	9 (45.0)	7 (35.0)	—
** **N2	42 (14.3)	67 (22.4)	—	12 (18.2)	15 (22.7)	—	3 (15.0)	6 (30.0)	—
** **NX	2 (0.68)	0 (0.00)	—	—	—	—	—	—	—
M stage	—	—	0.692	—	—	1.000	—	—	0.016
** **M0	223 (76.1)	218 (72.9)	—	0 (0.00)	1 (1.52)	—	18 (90.0)	10 (50.0)	—
** **M1	38 (13.0)	44 (14.7)	—	57 (86.4)	57 (86.4)	—	2 (10.0)	10 (50.0)	—
** **MX	29 (9.90)	31 (10.4)	—	9 (13.6)	8 (12.1)	—	—	—	—
Clinical stage	—	—	0.777	—	—	0.735	—	—	0.522
** **I	48 (16.4)	53 (17.7)	—	7 (10.6)	10 (15.2)		5 (25.0)	4 (20.0)	—
** **II	115 (39.2)	102 (34.1)	—	27 (40.9)	24 (36.4)		2 (10.0)	6 (30.0)	—
** **III	82 (28.0)	90 (30.1)	—	16 (24.2)	19 (28.8)		5 (25.0)	3 (15.0)	—
** **IV	38 (13.0)	44 (14.7)	—	16 (24.2)	13 (19.7)		8 (40.0)	7 (35.0)	—
KRAS mutation	—	—	0.667	—	—	—	—	—	—
** **No	17 (5.80)	14 (4.68)	—	—	—	—	—	—	—
** **Yes	16 (5.46)	13 (4.35)	—	—	—	—	—	—	—

### *HMMR* Levels in Blood Samples

**FIGURE 3:**
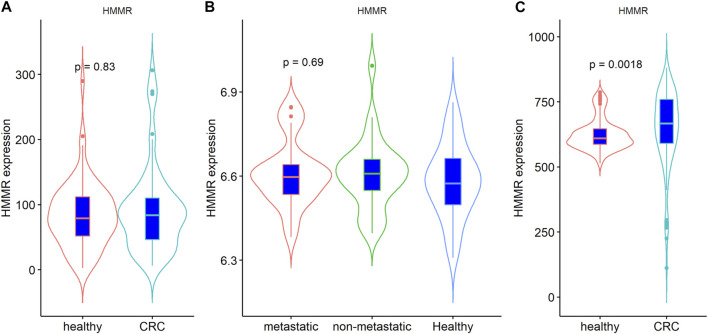
Comparison of blood levels of *HMMR* in CRC and healthy controls: **(A)** results of GSE164191; **(B)** results of GSE47756, including patients with metastatic and non-metastatic cancer; **(C)** results of clinical blood samples.

Furthermore, to explore the expression of *HMMR* in peripheral blood, two GEO datasets (GSE164191 and GSE47756) analyzing the expression of *HMMR* in peripheral blood were included in the analysis, and the blood samples of 120 patients with CRC and 100 healthy controls were collected and tested by ELISA. As shown in Figures 3A, B, HMMR levels in blood samples were not significantly different between patients with CRC and healthy controls in the GSE164191 and GSE47756 datasets; but *HMMR* levels, analyzed by ELISA, were significantly elevated in blood samples from patients with CRC than in those from healthy controls (Figure 3C, *p* < 0.01).

Next, we examined the association between *HMMR* levels and clinical parameters in CRC patients, as listed in Table 2, using median *HMMR* levels as the cutoff value. No significant differences in patients’ age, sex, TNM stage, clinical stage, tumor location, and the four tumor biomarkers (CEA, CA125, CA153, and CA199) (*p* < 0.05) were observed between samples with high and low *HMMR* levels; this suggested that HMMR levels in the peripheral blood of patients with CRC were independent of the clinical parameters and tumor biomarkers.

**TABLE 2:** Association of blood levels of HMMR with the clinical parameters.

	High (58)	Low (58)	*p*-Value
Age	56.7 ± 13.2	59.9 ± 12.0	0.187
Gender	—	—	0.866
** **Female	25 (43.1)	19 (39.6)	—
** **Male	33 (56.9)	29 (60.4)	—
Location	—	—	1.000
** **Colon	31 (53.4)	25 (52.1)	—
** **Rectal	27 (46.6)	23 (47.9)	—
T stage	—	—	0.617
** **T2	3 (5.17)	5 (10.4)	—
** **T3	20 (34.5)	14 (29.2)	—
** **T4	35 (60.3)	29 (60.4)	—
N stage	—	—	0.837
** **N0	14 (24.1)	13 (27.1)	—
** **N1	2 (3.45)	2 (4.17)	—
** **N2	41 (70.7)	31 (64.6)	—
** **NX	1 (1.72)	2 (4.17)	—
M stage	—	—	0.958
** **M0	47 (81.0)	40 (83.3)	—
** **M1	11 (19.0)	8 (16.7)	—
CEA	9.79 ± 28.9	6.38 ± 10.7	0.408
CA125	18.0 ± 18.0	21.4 ± 47.9	0.639
CA153	12.3 ± 5.40	10.5 ± 6.76	0.144
CA199	35.2 ± 132	30.5 ± 61.9	0.810

### Survival Analysis of *HMMR* in Patients With CRC

**FIGURE 4:**
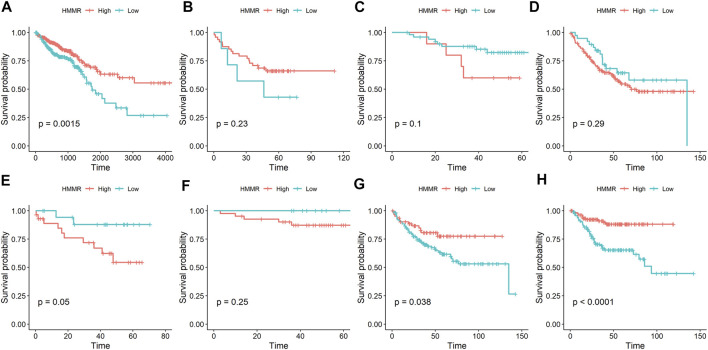
Association of the expression of *HMMR* with survival in patients with CRC. **(A)** OS results in TCGA-COAD and -READ datasets; **(B)** OS results in the GSE17537 dataset; **(C)** OS results in the GSE12945 dataset; **(D)** OS results in the GSE17536 dataset; **(E)** DFS results in the GSE17537 dataset; **(F)** DFS results in the GSE12945 dataset; **(G)** DFS results in the GSE17536 dataset; **(H)** DFS results in the GSE14333 dataset. OS: overall survival; DFS: disease-free survival.

To determine the prognostic value of *HMMR* in patients with CRC, we analyzed the overall survival (OS) and disease-free survival (DFS) using data from TCGA and four GEO datasets (GSE17537, GSE12945, GSE17536, and GSE14333). As shown in Figures 4A–D, low expression of *HMMR* indicated poor survival rate in TCGA; but there was no significant association between *HMMR* expression and OS in patients with CRC in three GEO datasets. Furthermore, analyses of two GEO datasets (GSE17536 and GSE14333) indicated a significant association between *HMMR* expression and DFS in patients with CRC, although the other two datasets (GSE17537 and GSE12945) were inconsistent with these results (Figures 4E–H). Taken together, these results fail to confirm the association between *HMMR* expression and survival in patients with CRC.

## Discussion

In the present study, we comprehensively analyzed the expression of HMMR in multiple types of CRC sample tissues, blood, and cell lines and in sequence datasets. The results indicated that the expression of *HMMR* mRNA was significantly increased in CRC tissues than in corresponding control tissues. However, the blood levels of *HMMR* were not significantly different between patients with CRC and healthy controls. Moreover, the clinical analysis failed to reveal the association of *HMMR* expression in tissues and peripheral blood with the TNM stage and clinical stage, suggesting that *HMMR* expression was independent of the progression of CRC. Furthermore, although the *HMMR* mRNA level was elevated in HT-29 cells than in normal colon mucosal epithelial cells, there was considerably decreased expression in the other three CRC cell lines, highlighting the variable expression of *HMMR* mRNA in CRC. Finally, analyses of datasets from TCGA and GEO failed to confirm the prognostic value of *HMMR* in OS and DFS in patients with CRC, suggesting that the predictive role of *HMMR* in CRC prognosis remains to be verified.

The molecular structure and biological function of *HMMR* have been documented in previous studies. Evidence suggests that the *HMMR* is a hydrophilic protein that lacks hydrophobic signal peptides or potential hydrophobic transmembrane domains (Choi et al., 2019); it also lacks the structural properties required for canonical extracellular export (Kouvidi et al., 2014). The expression of *HMMR* is critical for orientation of the mitotic spindle in human mitotic cells and is regulated by proliferation-associated transcription factors or signaling pathway members, such as FOXM1, E2F4, MYC (Lachmann et al., 2018), and Hippo (Wang et al., 2014). A study also revealed that the expression of HMMR is transcriptionally downregulated by the tumor suppressor p53 (Sohr and Engeland, 2008). The presence of *HMMR*-positive CRC cells is significantly associated with poor survival outcomes, independent of the TNM stage and adjuvant therapy (Koelzer et al., 2015). These findings suggest that the *HMMR* plays a critical role in the pathogenesis of CRC.

The expression of *HMMR* was reported to be upregulated in several cancer types (Shigeishi et al., 2014; Singleton, 2014; Buttermore et al., 2017), but it was also downregulated in other cancer types than in normal controls based on the results from the LAML and TGCT projects. In the present study, we found that the *HMMR* was significantly upregulated in gastrointestinal tract cancers, and results from TCGA and GEO datasets and clinical samples further validated the elevated expression of *HMMR* in CRC tissues. To determine the role of *HMMR* in the development of CRC, we analyzed the association between the expression of *HMMR* and clinical parameters. However, the results from the two large cohorts failed to reveal such associations. Additionally, the experimental analyses also confirmed only small associations, which indicated that the expression of *HMMR* in CRC tissues was independent of the tumor stage. Moreover, the expression of *HMMR* varied significantly between different CRC cell lines. These results demonstrated that the expression of *HMMR* was different in the tissues and cell lines.

As compared to the tissue samples, the role of *HMMR* levels in the blood of patients is more interesting to clinicians. Therefore, we examined *HMMR* levels in the blood of patients with CRC. However, the expression of *HMMR* in the blood was not significantly different between patients with CRC and healthy controls. The ELISA indicated significant differences in the expression of *HMMR* between patients with CRC and healthy controls; but considering the relatively small sample size, these results still need validation. Additionally, similar to the results from tissues, HMMR levels in the blood were not significantly associated with the clinical parameters, including the TNM stage.

A previous study suggested that high expression of *HMMR* was associated with poor survival in patients with CRC (Zlobec et al., 2008a). Another study showed similar results using immunohistochemistry (Zlobec et al., 2008b). However, in the present study, the OS and DFS in four GEO datasets were inconsistent, and those from TCGA were even opposite to the results of a previous study. Moreover, the association between *HMMR* and DFS was not significant in the three datasets. These discrepancies may be due to differences in treatment after diagnosis of CRC or the different detection methods of *HMMR*. Therefore, the prognostic value of *HMMR* in CRC remains to be validated in a larger cohort using a consistent detection method.

Previous studies have reported the expression of *HMMR* in CRC (Karamitopoulou et al., 2011; Mele et al., 2017); however, due to the limited number or types of samples, the clinical and prognostic significance of *HMMR* in CRC remains to be elucidated. The present study, as opposed to previous studies, included many types of samples with larger sample sizes and, thus, achieved more reliable results. However, some limitations of this study should be noted. First, several confounders, such as gene mutation and microsatellite instability status, may have affected the gene expression and prognosis of patients; but we could not analyze the effect of these factors on the expression of *HMMR* due to the limited data. Second, the number of clinical samples was relatively small, and the results still need to be verified in a larger cohort. Third, the mechanism of action of *HMMR* in CRC pathogenesis needs to be explored *in vivo* and *in vitro*.

## Conclusion

By comprehensively analyzing the sequencing and microarray data, clinical samples, blood samples, and cell lines, the present study demonstrated that the expression of *HMMR* was increased in CRC tissues but not in the blood. Moreover, the expression of *HMMR* was independent of CRC development and had no prognostic significance in patients with CRC.

## Data Availability

The raw data supporting the conclusions of this article will be made available by the authors, without undue reservation.
